# Combinatorial Mutagenesis and Selection to Understand and Improve Yeast Promoters

**DOI:** 10.1155/2013/926985

**Published:** 2013-06-06

**Authors:** Laila Berg, Trine Aakvik Strand, Svein Valla, Trygve Brautaset

**Affiliations:** ^1^Department of Biotechnology, Norwegian University of Science and Technology, Sem Sælands vei 6, 7491 Trondheim, Norway; ^2^Department of Molecular Biology, SINTEF Materials and Chemistry, Sem Sælands vei 2, 7465 Trondheim, Norway

## Abstract

Microbial promoters are important targets both for understanding the global gene expression and developing genetic tools for heterologous expression of proteins and complex biosynthetic pathways. Previously, we have developed and used combinatorial mutagenesis methods to analyse and improve bacterial expression systems. Here, we present for the first time an analogous strategy for yeast. Our model promoter is the strong and inducible *P*_*AOX*1_ promoter in methylotrophic *Pichia pastoris*. The Zeocin resistance gene was applied as a valuable reporter for mutant *P*_*AOX*1_ promoter activity, and we used an episomal plasmid vector to ensure a constant reporter gene dosage in the yeast host cells. This novel design enabled direct selection for colonies of recombinant cells with altered Zeocin tolerance levels originating solely from randomly introduced point mutations in the *P*_*AOX*1_ promoter DNA sequence. We demonstrate that this approach can be used to select for *P*_*AOX*1_ promoter variants with abolished glucose repression in large mutant libraries. We also selected *P*_*AOX*1_ promoter variants with elevated expression level under induced conditions. The properties of the selected *P*_*AOX*1_ promoter variants were confirmed by expressing luciferase as an alternative reporter gene. The tools developed here should be useful for effective screening, characterization, and improvement of any yeast promoters.

## 1. Introduction

Microorganisms are widely used in biotechnology as cell factories for sustainable production of proteins, biopolymers, and chemicals for medical, pharmaceutical and industrial applications. Today, bioprospecting programs exploring microbial diversity around the world lead to the identification of new species, new genes, and new products. As the majority of natural microorganisms are not cultivable under laboratory conditions, the application of robust microbial hosts for heterologous expression of genes and gene clusters is crucial both to understand microbial genetic diversity as well as to develop new value-added products. Access to well-defined and specialized promoter systems is important to enable functional expression and concomitant verification of the collected genes and pathways. We have previously developed combinatorial mutagenesis and selection methods to improve bacterial promoter systems for diverse purposes. In particular, we have worked with the strong and flexible *xylS/Pm* regulator/promoter system originating from TOL plasmid pWWO of *Pseudomonas putida* [1]. XylS belongs to the AraC-XylS family of transcription factors, and XylS can upon binding of effector molecules (alkylbenzoates) bind upstream of *Pm* and activate transcription (for a review, see [2]). We have constructed expression vectors by combining *xylS/Pm* with the minimal replicon *trfA/oriV* of the natural broad host range plasmid RK2 and demonstrated industrial level production of several medical recombinant proteins in *Escherichia coli* under high-cell-density cultivations [3, 4]. By using combinatorial mutagenesis and selection methods we have selected variants of the *Pm* promoter with improved expression properties in *E. coli*, and we have also shown that such methods are highly useful to generate new basic knowledge of bacterial gene expression [5–7]. The major principle of this approach was on one hand to generate large and mutant promoter sequence libraries and on the other hand to establish a direct selection method enabling efficient screening of the libraries for promoter variants with the desired improved properties. The selection system was based on using the *bla* gene (encoding the ampicillin-inactivating enzyme *β*-lactamase) as reporter placed under the transcriptional control of the *Pm* promoter on a plasmid, enabling direct selection of recombinant *E. coli* host cells with altered ampicillin tolerance levels. 

Besides bacteria, yeast is an important and sometimes a preferred host for recombinant expression of genes and pathways. The methylotrophic yeast *Pichia pastoris* has become an extensively used expression host, and the strong and inducible *P*_*AOX*1_ promoter is one of the most frequently applied promoters for gene expression in this organism [8–11]. Expression from the *P*_*AOX*1_ promoter is tightly regulated by the present carbon source, involving strong repression in the presence of glucose or glycerol and full induction by methanol when the repressive carbon sources are absent [12, 13]. Yeast promoters are in general longer and more complex than bacterial promoters, and the understanding of the about 950 base pairs long *P. pastoris P*_*AOX*1_ promoter DNA region is limited, both with regard to its *cis*-acting elements and the molecular mechanisms underlying its regulation. In recent years, several attempts have been made to understand and improve the *P*_*AOX*1_ promoter by using rational design and site-directed mutagenesis [14–16]. In spite of considerable efforts, the details about the *cis*-acting elements involved in the glucose regulation of the *P*_*AOX*1_ promoter and the underlying molecular mechanisms are still not known. Also, the improvements of the induced expression level in these reports were moderate. This emphasizes the need for effective methods to analyse and improve yeast promoter systems. 

In the present study, we introduce a novel combinatorial mutagenesis and selection strategy in yeast by using *P. pastoris* and the *P*_*AOX*1_ promoter as model system. We demonstrate for the first time that this approach also works in yeast and that it can be used both to alter the regulation properties as well as to improve the maximized expression level of the *P*_*AOX*1_ promoter. The methodology presented here should be valuable both to manipulate and improve yeast promoters, for promoter probe approaches, and for applied perspectives as well as to generate basic understanding of microbial promoters.

## 2. Materials and Methods 

### 2.1. Microbial Strains and Growth Media

Strains and vectors used in this study are listed in Table 1, and primers used for the vector constructions are listed in Table 2. *E. coli* cells were generally grown at 37°C in L broth (1% tryptone, 0.5% yeast extract, and 0.5% NaCl) or on L agar (L broth with 2% agar). When Zeocin was used for selection, the pH in the L broth and agar was adjusted to 7.5. Ampicillin (100 *μ*g/mL), kanamycin (50 *μ*g/mL), and Zeocin (25 *μ*g/mL) were added as appropriate.

*P. pastoris* cells were generally grown at 30°C in rich media with glucose (YPD; 2% peptone, 1% yeast extract, and 2% glucose) or on YPD agar (YPD broth with 2% agar). YPDS agar (YPD agar with 1 M sorbitol) was used for transformation purposes. The minimal media contained 1.34% yeast nitrogen base, 4 · 10^−5^% biotin, 0.004% histidine, and 0.5% methanol. 2% agar was included for agar media preparation. Methanol (0.5%) was added once a day to minimal media containing methanol to maintain the induction, for agar media this was obtained by adding 150 *μ*L (9 cm plates) or 500 *μ*L (14 cm plates) methanol to the lid of inverted plates. G418 (500 *μ*g/mL) was added as appropriate.

### 2.2. Standard DNA Manipulations

A RbCl protocol (http://www.promega.com/) was used for transformation of *E. coli*. Plasmids were isolated from *E. coli* by using WizardPlus SV minipreps DNA purification kit (Promega) or Qiafilter maxi plasmid purification kit (Qiagen), and enzymatic manipulations were performed as described by the manufacturers. PCR reactions were performed using the Expand high-fidelity PCR system kit (Boehringer Mannheim) for cloning purposes and the PfuUltra II Fusion HS DNA polymerase (Strategene) for site-directed mutagenesis applications. Sequencing analyses were purchased from Eurofins MWG operon (http://www.eurofinsdna.com/).

For transformation of yeast, in general 10 *μ*L of circular plasmid preparation was transformed to *P. pastoris* in accordance with the electroporation protocol from Invitrogen (http://www.Invitrogen.com/). Plasmids were isolated from *P. pastoris* by using the Easy Yeast Plasmid Isolation kit (Clontech).

### 2.3. Agar Plate Assay for Determination of the Maximal Zeocin Tolerance Levels of the *P. pastoris* Host Cells

An agar plate assay was used to indirectly determine the yeast promoter activity as the maximal Zeocin tolerance levels of the *P. pastoris* host cells. This is a modified version of the previously used agar plate assay developed for the indirect determination of the *Pm* promoter activity in *E. coli* as maximal ampicillin resistance levels of the host cells [17]. In detail, freshly transformed *P. pastoris* GS115 strains were inoculated into 100 *μ*L YPD broth in 96 micro-well plates and incubated at 30°C with shaking overnight. Next, the cells were diluted with a 96-pin replicator in 0.9% NaCl (100 *μ*L) and plated on YPD agar or minimal agar media with methanol containing various Zeocin concentrations. Usually, the cells were also plated on YPD agar containing G418. The plates were incubated at 30°C for three days. The maximal Zeocin tolerance level determinations were carried out with minimum four technical recurrences.

### 2.4. Construction of Promoter Mutant Libraries

The mutant libraries were established and stored as plasmid libraries in *E. coli* DH5*α*, and circular plasmids were transformed to *P. pastoris* GS115 (see below). In total, two different mutant libraries were made in the *P*_*AOX*1_ promoter with the *Zc* ^*R*^ gene as the promoter reporter. The presence of both a kanamycin- and an ampicillin resistance gene allows for selection of the plasmid libraries in *E. coli,* and the kanamycin resistance gene also confers resistance against the antibiotic G418 in *P. pastoris*, avoiding that *P*_*AOX*1_ expression levels (Zeocin resistance) could affect the composition of the libraries in any way. 

Plasmid pPPE20 was used when constructing the mutant library in the promoter core region of the *P*_*AOX*1_ promoter (plasmid library LC). The construction of the LC plasmid library is similar to the procedure previously described for the *Pm* promoter libraries [5, 6]. The oligonucleotides were designed to constitute a double-stranded DNA fragment containing the *P*_*AOX*1_ promoter core region with EcoRI- and NheI-compatible ends when annealed for subsequent easy cloning into the pPPE20 vector. One of the oligonucleotides corresponded to the wild-type sequence (5′-CTAGCAGTCGCAATTATGAAAGTAAGCTAATAATGATGATAAAAAAAAAGGTTTAAGACAGGGCAGCTTCCTTCTGTTTATATATTGCTGTCAAGTAGG-3′), and the oligonucleotide corresponding to the complementary strand was randomly mutagenized by the use of a doped oligonucleotide solution (5′-AATTC123122431341332323233313433443341241112421223331122222222232132132232234122312221323322414312G-3′, where the numbers in the sequence indicate the doping percentages of the nucleotides: 1 = 79% C, 7% A, 7% T, and 7% G; 2 = 79% T, 7% A, 7% C, and 7% G; 3 = 79% A, 7% C, 7% T, and 7% G; 4 = 79% G, 7% A, 7% T, and 7% C). Approximately 410000 *E. coli* DH5*α* transformants were obtained, constituting the LC plasmid library.

Plasmid pPPE50 was used when constructing the plasmid library LU, and the construction of this library is identical to that of the LC plasmid library, except that the oligonucleotides were designed to constitute a double-stranded DNA fragment containing the *P*_*AOX*1_ promoter core upstream region with KpnI- and EcoRI-compatible ends when annealed for subsequent easy cloning into the pPPE50 vector. As before, one of the oligonucleotides corresponded to the wild-type sequence (5′-AATTCGAACAGTTAAATTTTGATCATTAACGTTAGGCTATCAGCAGTATTCCCACCAGAATCTTGGAAGCATACAATGTGGAGACAATGCATAAGGTAC-3′), and the oligonucleotide corresponding to the complementary strand was randomly mutagenized by the use of a doped oligonucleotide solution (5′-C22324132242121131322423241221133432212442444332312412432341123314223324321333322233124221G-3′, where the numbers in the sequence indicate the doping percentages of the nucleotides: 1 = 88% C, 4% A, 4% T, and 4% G; 2 = 88% T, 4% A, 4% C, and 4% G; 3 = 88% A, 4% C, 4% T, and 4% G; 4 = 88% G, 4% A, 4% T, and 4% C). Approximately 1 million *E. coli* DH5*α* transformants were obtained, constituting the LU plasmid library.

### 2.5. Screening for *P*_*AOX*1_ Promoter Variants

The Qiafilter maxi plasmid isolation kit (Qiagen) was used for high-yield purification of the plasmid libraries from *E. coli* DH5*α* according to the procedure described by the manufacturers in order to obtain sufficient transformation efficiencies of these libraries into *P. pastoris*. Circular plasmids were transformed to *P. pastoris* GS115 and the transformation suspensions were plated on rich agar media with glucose or minimal agar media with methanol containing increasing concentrations of Zeocin to directly select for the transformants containing the *P*_*AOX*1_ promoter variants leading to stimulated expression compared to that of the wild-type. Normally, 100 *μ*L of the transformation suspension was plated on 9 cm agar plates, except for the big-scale screening experiment of the LC plasmid library on rich agar media with glucose (plating above 100000 transformants) in which 2.1 mL of the transformation suspension was plated on square Corning bioassay plates (24.5 × 24.5 cm). To determine an approximate number of the transformants containing the plasmid that were plated, the transformation suspension was also plated on YPDS agar containing G418. 

### 2.6. Luciferase Activity Assay

Freshly transformed *P. pastoris* GS115 strains were grown according to the protocols for induced expression of the *P*_*AOX*1_ promoter by Invitrogen (http://www.invitrogen.com/). For repressed expression, freshly prepared *P. pastoris* GS115 transformants were inoculated to 10 mL YPD in 125 mL bottles and incubated at 225 rpm and 30°C overnight. Next, overnight cultures were inoculated to 50 mL YPD in 250 mL baffled bottles to a final optical density at 600 nm of 0.008 and incubated at 225 rpm and 30°C for 3 days. Samples were collected as indicated in the results. Lysis of the cell samples was carried out according to the protocols by Invitrogen and, the luciferase activity assay was performed as described by the manufacturers (http://www.promega.com/). The luciferase activity analyses were repeated at least twice, and measurements were carried out with minimum three technical recurrences. 

## 3. Results and Discussion

### 3.1. Design and Construction of the Cassette Expression Plasmid pPPE17 to Enable Direct Selection of Yeast Promoter Variants with Altered Expression Properties

**Figure 1:**
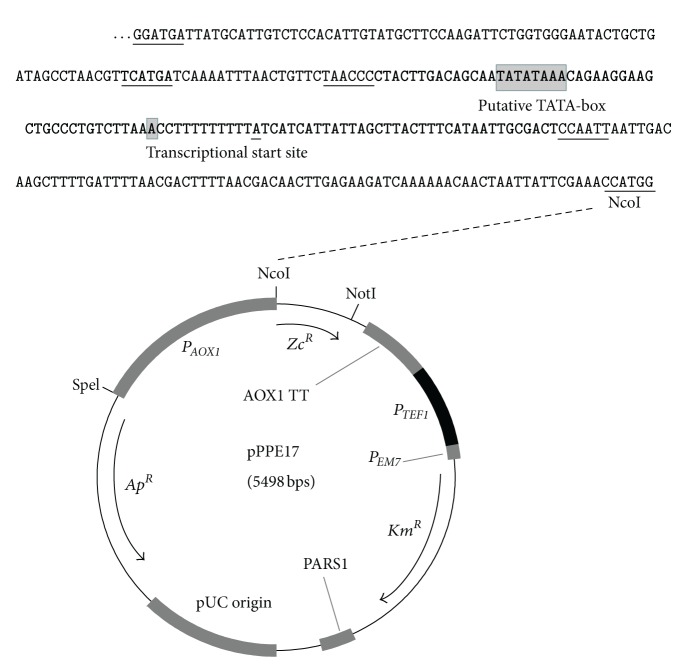
Physical map of the *E. coli*-*P*. *pastoris* shuttle plasmid pPPE17 and its derivatives. The restriction enzyme sites shown are unique. Abbreviations: *P*_*AOX*1_: *P. pastoris* AOX1 promoter; *Zc* ^*R*^: Zeocin resistance gene; AOX1 TT: *P. pastoris* AOX1 transcription termination region; *P*_*TEF*1_: yeast promoter TEF1; *P*_*EM*7_: bacterial promoter EM7; *Km* ^*R*^: kanamycin resistance gene (confers resistance against kanamycin in bacteria and G418 in yeast); PARS1: *P. pastoris* autonomous replication sequence; pUC origin: bacterial replication sequence; *Ap* ^*R*^: ampicillin resistance gene (*bla*). DNA sequence of the 3′-part of the wild-type *P*_*AOX*1_ promoter (base pairs 670 to 950) is displayed above the plasmid map. The promoter core region is shown in bold (see also text). The transcriptional start site (A) and the putative TATA-box (TATATAAA) are highlighted in grey. The BspHI (TCATGA) restriction enzyme site is written in bold and underlined and is deleted by the introduction of a point mutation (T**A**ATGA) in plasmids pPPE20 and pPPE50. GGATGA is mutated to the KpnI (GGTACC) restriction enzyme site in plasmids pPPE50. TAACCC is mutated to the EcoRI (GAATTC) restriction enzyme site in plasmids pPPE20 and pPPE50. CCAATT is mutated to the NheI (GCTAGC) restriction enzyme site in plasmid pPPE20 and pPPE50.

It has been well documented that *xylS/Pm* is a strong and flexible promoter system useful for high-level, as well as for controlled, recombinant expression of genes and pathways in Gram-negative bacteria (see Section 1). Still, *Pm* promoter variants with new and improved properties have been generated by using combinatorial mutagenesis and in such approaches we have taken advantage of the correlation between the expression level of the *bla* gene (encoding *β*-lactamase) and the corresponding ampicillin tolerance level of the host cells [5, 6, 18]. We here chose to apply the Zeocin resistance gene *Zc* ^*R*^ as reporter due to its documented usefulness in identifying yeast transformants with multicopy chromosomal insertions based on the Zeocin tolerance levels of the host cells (http://www.invitrogen.com/). When using this strategy to directly select recombinant yeast strains with altered Zeocin tolerance levels, it is critical to ensure a constant *Zc* ^*R*^ gene dosage in the yeast host cells. Therefore, we chose to use the *P. pastoris* episomal (i. e. autonomously replicating) plasmid vector pBGP1 [19] as the carrier for the expression cassette, in favour of using chromosomally integrated vectors which may lead to different expression levels depending on the location of the integration and possible multicopy insertions of the expression cassette. Plasmid pBGP1 was used as basis to generate the pPPE17 plasmid, which contains the *Zc* ^*R*^ gene under control of the *P*_*AOX*1_ promoter. This design enables the substitution of the yeast promoter and/or gene of interest in a one-step cloning procedure (Figure 1). In addition, plasmid pPPE22 was constructed by substituting the *P*_*AOX*1_ promoter in pPPE17 with the constitutive *P. pastoris P*_*GAP*_ promoter (see Figure 1), and it was included as a valuable control system. The latter promoter is reported to give strong expression under both the induced (presence of methanol and absence of repressive carbon sources) and repressed (presence of glucose) growth conditions for the *P*_*AOX*1_ promoter [20]. 

Both plasmids pPPE17 and pPPE22 were transformed into host strain *P. pastoris* GS115, and the maximal Zeocin tolerance levels were determined under *P*_*AOX*1_-promoter-repressed conditions for strains GS115, GS115 (pPPE17), and GS115 (pPPE22). We chose to use rich agar media with glucose (YPD) for the repressed growth condition purpose. Recombinant strains GS115 (pPPE17) and GS115 (pPE22) displayed about 5-fold and 400-fold higher Zeocin tolerance levels (25 *μ*g/mL and 2000 *μ*g/mL, resp.) compared to that of host GS115 (up to 5 *μ*g/mL). The results show that the expression level from the *P*_*AOX*1_ promoter is low under the glucose-repressed conditions tested, as expected. Further, the results demonstrate a correlation between the expected expression levels of the *Zc* ^*R*^ gene from the two promoters and the tolerance levels of the corresponding host cells. 

Plasmid pPPE17 was further modified into a promoter-cloning cassette system by deletion of a BspHI restriction enzyme recognition site and insertion of two unique restriction enzyme recognition sites (EcoRI and NheI) in the *P*_*AOX*1_ promoter, generating plasmid pPPE20 (Figure 1). These modifications did not affect the expression properties of the plasmid compared to pPPE17 (data not shown), and pPPE20 was used for screening of *P*_*AOX*1_ promoter mutant libraries for variants with altered expression properties as described next.

### 3.2. Selection of *P*_*AOX*1_ Promoter Variants with Abolished Glucose Repression

The *P*_*AOX*1_ *cis*-acting promoter elements involved in the glucose repression are not yet identified (see Section 1). We here initially selected for *P*_*AOX*1_ promoter variants with altered glucose repression to establish and assess the combinatorial mutagenesis approach. Previous mutagenesis strategies aiming at identifying *P*_*AOX*1_ promoter variants with altered glucose repression have focused on the promoter region upstream of the putative TATA-box [10, 12]. We here decided to explore the impact on the glucose regulation of two distinct regions of the *P*_*AOX*1_ promoter. First, the *P*_*AOX*1_ promoter core region was here defined as the region from about 15 base pairs upstream of the putative TATA-box to about 35 base pairs downstream of the transcriptional start site (see Figure 1). A mutant library was constructed in this *P*_*AOX*1_ promoter core region by the use of randomly mutated synthetic oligonucleotides (designated plasmid library LC), cloned into the NheI/EcoRI sites of pPPE20 (Figure 1) and transformed into *P. pastoris* GS115. Second, we constructed a promoter mutant library (designated plasmid library LU) by randomizing the 90 base pairs region directly upstream of the *P*_*AOX*1_ promoter core region, corresponding to the region that was extensively studied by Hartner et al. [14] by using rational design. To accomplish this, plasmid pPPE20 was modified by the insertion of a unique restriction enzyme site (KpnI) in the *P*_*AOX*1_ promoter, generating plasmid pPPE50. In the latter plasmid, the promoter upstream region can be easily substituted by a one-step cloning procedure using the unique EcoRI/KpnI restriction sites (Figure 1). As for pPPE20 (see above), this modification did not affect the expression properties of pPPE50 compared to pPPE17 (data not shown).

**Figure 2:**
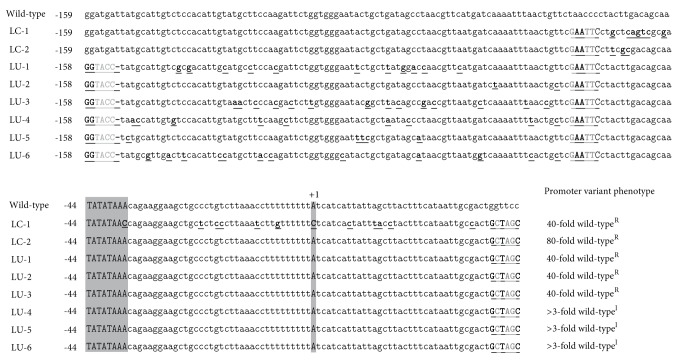
DNA sequence of region between base pairs-159 to +41 (promoter core region and the about 90 base pairs region directly upstream of the promoter core region, the transcriptional start site is set as +1) of the wild-type *P*_*AOX*1_ promoter and variants LC-1, LC-2, and LU-1 to LU-6. Mutations are shown in bold and underlined. Deletion mutations are indicated by short horizontal lines. The transcriptional start site (A) and the putative TATA-box (TATATAAA) are written in upper case and highlighted in grey. Mutations leading to restriction-enzyme-site insertions or deletions without affecting the promoter activity are written in grey. The KpnI (GGTACC), EcoRI (GAATTC), and NheI (GCTAGC) restriction enzyme sites are written in upper case and underlined. The relative Zeocin tolerance levels of the promoter variants compared to the wild-type promoter under repressed (wild-type^R^) and induced (wild-type^I^) growth conditions are shown.

The LC and LU plasmid libraries were then screened for increased Zeocin tolerance levels relative to the control strain *P. pastoris* GS115 (pPPE17) under glucose-repressed conditions. Over 100000 transformants representing library LC and over 5000 transformants representing library LU were screened, and five totally different *P*_*AOX*1_ promoter variants (named LC-1, LC-2, LU-1, LU-2, and LU-3) with strongly elevated Zeocin tolerance levels were isolated. The selected promoter variants provided significantly higher maximal Zeocin tolerance levels of the GS115 cells under repressed conditions (up to 2000 *μ*g/mL) compared to that of the wild-type *P*_*AOX*1_ promoter (25 *μ*g/mL). The five selected promoter variants had between 2 (LU-2) and 18 (LC-1) point mutations (Figure 2). The LU-2 and the LC-2 promoter variants have only two and three point mutations, respectively, suggesting that single nucleotides directly upstream of the TATA box are important for glucose repression of this promoter. The LC-1, LU-1, and LU-3 promoter variants, on the other hand, have multiple (between 11 and 19) point mutations distributed over the entire randomized promoter regions and thus both upstream and downstream of the TATA-box. Together, these data may suggest the existence of several *cis*-acting promoter elements involved in the glucose repression of the *P*_*AOX*1_ promoter. These results demonstrated that the combinatorial mutagenesis and selection strategy can be used to efficiently select for altered promoter variants, and they also show that the *P*_*AOX*1_ promoter core region and the 90 base pairs upstream region can have high impact on the glucose repression under the conditions tested.

### 3.3. Selected *P*_*AOX*1_ Promoter Variants Retain Their Characteristics When Expressing an Alternative Reporter Protein, the Firefly Luciferase

**Figure 3:**
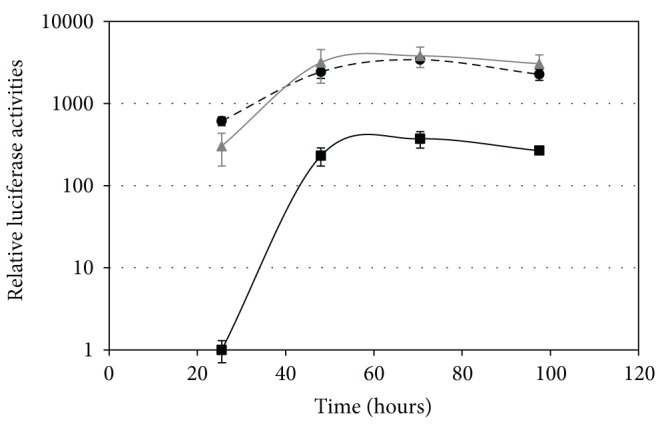
Relative luciferase activities under repressed growth conditions and during the glucose-depletion phase for the wild-type *P. pastoris P*_*AOX*1_ promoter (black squares) and variants LC-2 (grey triangles) and LU-2 (black circles, dashed line) in plasmid pPPE35 expressed in *P. pastoris* GS115. Enzyme activities are shown in logarithmic scale. The values are the average of two biological replicas, and the error bars show the standard deviations. The activities are relative to the wild-type, for which the value at the first time point is arbitrarily set to 1.

Previous combinatorial mutagenesis work with the bacterial *Pm* promoter has shown that there can be a strong context dependency between the promoter sequence and the coding region of the reporter gene which often affects the expression level in an unpredictable manner [5, 18]. Two of the selected *P*_*AOX*1_ promoter variants (LC-2 and LU-2) were therefore chosen for expression studies in liquid *P. pastoris* cultures with the alternative reporter gene *luc+*, encoding firefly luciferase. For this purpose, the *Zc* ^*R*^ gene in the pPPE17 plasmids containing the wild-type *P*_*AOX*1_ promoter and each of the LC-2 and LU-2 promoter variants was substituted with the *luc+* gene. The luciferase activities of GS115 cells harbouring these plasmids were quantified under repressed conditions and during the glucose-depletion phase in shake flasks (Figure 3). Both promoter variants LC-2 and LU-2 gave strongly enhanced luciferase activities at the first time point compared to that of the wild-type (about 300-fold and 600-fold, resp.). These differences decrease to around 10-fold for the remaining time points for both of the promoter variants (the second time point represents the transition from exponential to stationary growth phase). Thus, the increase in activity after the first time point is much larger for the wild-type, which shows that the LC-2 and LU-2 promoter variants are far less affected by the depletion of glucose compared to that of the wild-type promoter (Figure 3). Taken together, these results confirmed that LC-2 and LU-2 are *P*_*AOX*1_ promoter variants with abolished glucose repression irrespective of the context of the gene to be expressed. 

### 3.4. Selection of *P*_*AOX*1_ Promoter Variants with Increased Expression Level under Induced Conditions

Finally, we wanted to test if it was possible to select *P*_*AOX*1_ promoter variants with increased expression levels under induced conditions. As an initial test, the maximum Zeocin tolerance levels under induced conditions (minimal agar media with methanol) of strains GS115 and GS115 (pPPE17) were determined and found to be 25 *μ*g/mL and 2000 *μ*g/mL, respectively. Next, over 5000 transformants representing each of the LC and LU promoter libraries (see above) were screened with respect to increased Zeocin tolerance levels relative to GS115 cells harbouring original plasmid pPPE17 under *P*_*AOX*1_-induced conditions. In total, three promoter variants (LU-4, LU-5, and LU-6) were identified that caused increased Zeocin tolerance levels of the GS115 cells under induced conditions (7000 *μ*g/mL or more) compared to that of the wild-type promoter (2000 *μ*g/mL). The three selected promoter variants contained between 5 and 12 point mutations (Figure 2). These promoter variants contain multiple (between 5 and 12) point mutations distributed over the randomized region, analogous to the LU-1 and LU-3 variants that were screened for increased activity under glucose-repressed conditions (see above). The biological reason for these results is unclear. One potential explanation might be that there are *cis*-acting promoter elements important for both methanol induction and glucose repression located in the same region of the *P*_*AOX*1_ promoter.

For practical reasons we found that using Zeocin concentrations higher than 7000 *μ*g/mL was undesirable, and due to this the identified promoter variants were not further ranked at this stage. Instead, luciferase expression studies with GS115 cells under induced growth conditions were performed in shake flasks for two of these *P*_*AOX*1_ promoter variants (LU-4 and LU-5). Analyses of samples withdrawn at the time points for transition from exponential to stationary growth phase (about 72 hours) showed a similar activity for the LU-5 promoter variant and the wild-type promoter, whereas variant LU-4 displayed a 2-fold increase in luciferase activity compared to that of the wild-type (data not shown). This demonstrates that the combinatorial mutagenesis and selection strategy can be applied to also generate improved *P*_*AOX*1_ promoter variants under induced conditions. The potential is (further) likely to be greater than indicated here, considering that only a small fraction of the mutant libraries was screened and a narrow selection window was used (a concentration of Zeocin ranging from 2000 to 7000 *μ*g/mL).

## 4. Conclusions

Mutational analysis is an efficient approach to map specific promoter functions and properties to defined sequence motifs and nucleotides. Rational methods including site-directed mutagenesis are preferred but such approaches rely on a detailed preinsight into the relation between the sequence and function, which is typically not the case for most promoters. Accordingly, the need for creating libraries with random mutations distributed over defined regions might be preferred; however, such strategies are dependent on efficient selection and screening methods to identify mutations leading to the desired phenotypes. The current study demonstrates for the first time a combinatorial mutagenesis and selection approach for generating new understanding of the regulation of promoter systems and to develop improved expression systems in yeast. The tools and technology developed here should be valuable for investigations and improvement of any yeast promoter system, leading to new basic knowledge as well as to useful tools for heterologous expression of single genes and complex biosynthetic pathways in yeast. Understanding promoters is crucial to generate new basic knowledge of microbial gene expression and regulation at all levels, and development of new and specialized expression systems is important to experimentally analyze microbial diversity including metagenome libraries for new functions and products. Finally, the tools and technology developed here should also be useful as a promoter probe system for efficient screening and characterization of natural promoter libraries in yeast.

## Figures and Tables

**Table 1:** Strains and vectors used in this study.

Strain or plasmid	Description^a,b,c^	Reference or source
*E.coli* strains		
DH5*α*	General cloning host.	BRL
ER2925	Host used for cloning experiments involving methylation-sensitive restriction enzymes.	NEB
*P. pastoris* strain		
GS115	Host used for all of the *P. pastoris* screening and growth experiments.	Invitrogen
Vectors		
pIB11	Bacterial RK2-based expression vector harboring *Pm/xylS* regulatory promoter system with the *bla* gene as reporter, Km^R^.	[6]
pGL3-control vector	Firefly luciferase reporter vector, Ap^R^.	Promega
pBGP1	*P. pastoris* episomal expression vector harbouring the *P*_*GAP*_ promoter. *E. coli*-*P. pastoris* shuttle vector, Zc^R^, Ap^R^.	[19]
pPICZ A	*P. pastoris* expressional vector harbouring the *P*_*AOX*1_ promoter.* E. coli*-*P. pastoris* shuttle vector, Zc^R^.	Invitrogen
pPPE3	pBGP1 derivative in which the three BspHI restriction sites are deleted including one situated in the *P*_*GAP*_ promoter by using site-directed mutagenesis with primer pair 1, 2, and 3, Zc^R^, Ap^R^.	This study
pPPE8	pPPE3 derivative in which a BspHI restriction enzyme site was inserted in the start of the zeocin-resistance gene by using site-directed mutagenesis with primer pair 4, Ap^R^.	This study
pPPE10	pPPE8 derivative in which the zeocin-resistance gene was substituted with the kanamycin-resistance gene from plasmid pIB11. The *Km* ^*R*^ gene was amplified from pIB11 with primer pair 5 and end digested with BspHI and EcoRV prior to ligation into the corresponding sites of pPPE8, Ap^R^, Km^R^, G418^R^.	This study
pPPE11	pPPE10 derivative in which a NcoI restriction enzyme site was inserted in the start of the Alpha signal by using site-directed mutagenesis with primer pair 6, Ap^R^, Km^R^, G418^R^.	This study
pPPE12	pPPE11 derivative in which the zeocin-resistance gene was inserted as reporter for the modified *P*_*GAP*_ promoter. The zeocin-resistance gene was amplified from plasmid pBGP1 with primer pair 7 and end digested with NcoI and NotI prior to ligation into the corresponding sites of pPPE11, Ap^R^, Km^R^, G418^R^.	This study
pPPE17	pPPE12 derivative in which the modified *P*_*GAP*_ promoter was substituted with the *P*_*AOX*1_. The *P*_*AOX*1_ promoter was amplified from pPICZ A with primer pair 8 and end digested with SpeI and NcoI prior to ligation into the corresponding sites of pPPE12, Ap^R^, Km^R^, G418^R^.	This study
pIPA1	pPICZ A derivative in which the BspHI restriction enzyme site in the *P*_*AOX*1_ promoter is deleted by using site directed mutagenesis with primer pair 9, Zc^R^.	This study
pIPA4	pIPA1 derivative in which an EcoRI restriction enzyme site is inserted directly upstream of the putative TATA-box in the *P*_*AOX*1_ promoter by using site-directed mutagenesis with primer pair 10, Zc^R^.	This study
pIPA5	pIPA4 derivative in which a NheI restriction enzyme site is inserted downstream of the transcriptional start site in the *P*_*AOX*1_ promoter by using site-directed mutagenesis with primer pair 11, Zc^R^.	This study
pPPE20	Similar to pPPE17 except for that the *P*_*AOX*1_ promoter was substituted with the modified *P*_*AOX*1_ promoter from plasmid pIPA5. The *P*_*AOX*1_ promoter was amplified from plasmid pIPA5 with primer pair 12 and end digested with SpeI and NcoI prior to ligation into the corresponding sites of pPPE12, Ap^R^, Km^R^, G418^R^.	This study
pPPE22	Similar to pPPE17 except for that the *P*_*AOX*1_ promoter was substituted with the wild-type *P*_*GAP*_ promoter from plasmid pBGP1 by subcloning the SpeI/MfeI fragment from plasmid pBGP1 into the corresponding sites of plasmid pPPE12, Ap^R^, Km^R^, G418^R^.	This study
pPPE35	Similar to pPPE17 except for that the *Zc* ^*R*^ gene is substituted with the *luc+* gene (encoding firefly luciferase). The *luc+* gene was amplified from plasmid pGL3-control vector with primer pair 13 and end digested with NcoI and NotI prior to ligation into the corresponding sites of pPPE17, Ap^R^, Km^R^, G418^R^.	This study
pPPE50	Similar to pPPE20 except for that a KpnI restriction enzyme site is inserted in the *P*_*AOX*1_ promoter about 90 base pairs upstream of the EcoRI restriction enzyme site by using site-directed mutagenesis with primer pair 14, Ap^R^, Km^R^, G418^R^.	This study

^a^Ap: ampicillin; Km: kanamycin; Zc: Zeocin, G418: aminoglycoside antibiotic.

^b^Sequences of the primer pairs are listed in Table 2.

^c^The target region was subcloned and verified by sequencing for all of the site-directed mutagenesis applications. Also, all cloned regions amplified by PCR were verified by sequencing.

**Table 2:** Primers used in genetic modifications.

Primer pair number	Sequence^a^
1	5′-TGTATCCGCTAATGAGACAAT-3′ and 5′-TTGTCTCATTAGCGGATACA-3′
2	5′-GATTTTGGTAATGAGATTAT-3′ and 5′-ATAATCTCATTACCAAAATC-3′
3	5′-GGACGCATGTAATGAGATTATT-3′ and 5′-AATAATCTCATTACATGCGTCC-3′
4	5′-AGGAACTAAATCATGACCAAGTTGAC-3′ and 5′-GTCAACTTGGTCATGATTTAGTTCCT-3′
5	5′-TAATAATCATGAGCCATATTCAAC-3′ and 5′-TAGATATCATTAGAAAAACTCATC-3′
6	5′-ATTTCGAAACCATGGGATTTCCTTC-3′ and 5′-GAAGGAAATCCCATGGTTTCGAAAT-3′
7	5′-TAATAACCATGGCCAAGTTGACC-3′ and 5′-TAATAAGCGGCCGCATCAGTCCTGCTCCTC-3′
8	5′-TAATAAACTAGTAGATCTAACATCCAA-3′ and 5′-TAATAACCATGGTTTCGAATAATTAG-3′
9	5′-AGCCTAACGTTAATGATCAAAATT-3′ and 5′-AATTTTGATCATTAACGTTAGGCT-3′
10	5′-AAAATTTAACTGTTCGAATTCCTACTTGACAGCAA-3′ and 5′-TTGCTGTCAAGTAGGAATTCGAACAGTTAAATTTT-3′
11	5′-TCATAATTGCGACTGCTAGCAATTGACAAGCTTT-3′ and 5′-AAAGCTTGTCAATTGCTAGCAGTCGCAATTATGA-3′
12	5′-TAATAAACTAGTAGATCTAACATCCAA-3′ and 5′-TAATAACCATGGTTTCGAATAATTAG-3′
13	5′-TAATAACCATGGAAGACGCCAAAAACA-3′ and 5′-TAATAAGCGGCCGCATTACACGGCGATCTTTC-3′
14	5′-ACCCGCTTTTTGGTACCTTATGCATTGT-3′ and 5′-ACAATGCATAAGGTACCAAAAAGCGGGT-3′

^a^The restriction enzyme site is underlined.
